# 6-(1-Adamant­yl)-3-(2-chloro­phen­yl)-1,2,4-triazolo[3,4-*b*][1,3,4]thia­diazole

**DOI:** 10.1107/S1600536811011391

**Published:** 2011-03-31

**Authors:** M. Nawaz Tahir, Mahmood-ul-Hassan Khan, Shahid Hammed, Tanveer Hussain Bokhari, Saira Hina

**Affiliations:** a Department of Physics, University of Sargodha, Sargodha, Pakistan; b Department of Chemistry, Quaid-i-Azam University, Islamabad 45320, Pakistan; c Department of Chemistry, GC University, Faisalabad, Pakistan; d Department of Animal Sciences, Quaid-i-Azam University, Islamabad 45320, Pakistan

## Abstract

In the title compound, C_19_H_19_ClN_4_S, the 2-chloro­phenyl and [1,2,4]triazolo[3,4-*b*] [1,3,4]thia­diazole fragments (r.m.s. deviations of 0.015 and 0.017 Å, respectively) are oriented at a dihedral angle of 55.76 (6)°. The adamantane group exhibits extensive rotational disorder about the single C—C bond to the thia­diazole ring, which was modelled as occupying four orientations each with 0.25 occupancy. In the crystal, the chloro­phenyl rings exhibit π–π stacking inter­actions with centroid–centroid distances of 3.9526 (18) Å.

## Related literature

For background and the structure of the fluoro analogue, see: Khan *et al.* (2009 ▶).
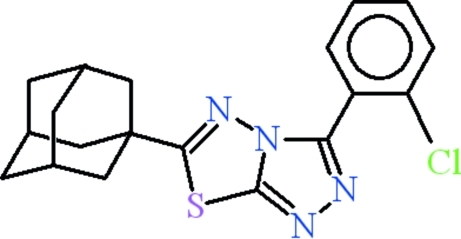


## Experimental

### 

#### Crystal data

C_19_H_19_ClN_4_S
*M* *_r_* = 370.89
Monoclinic, 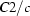

*a* = 24.5210 (12) Å
*b* = 9.1356 (5) Å
*c* = 19.1943 (16) Å
β = 124.282 (1)°
*V* = 3552.8 (4) Å^3^
*Z* = 8
Mo *K*α radiation
μ = 0.34 mm^−1^
*T* = 296 K
0.28 × 0.24 × 0.20 mm

#### Data collection

Bruker Kappa APEXII CCD diffractometer
Absorption correction: multi-scan (*SADABS*; Bruker, 2005 ▶) *T*_min_ = 0.945, *T*_max_ = 0.956
16675 measured reflections
3202 independent reflections
2676 reflections with *I* > 2σ(*I*)
*R*_int_ = 0.025

#### Refinement

*R*[*F* ^2^ > 2σ(*F* ^2^)] = 0.048
*wR*(*F* ^2^) = 0.125
*S* = 1.02
3202 reflections
257 parameters
84 restraints
H-atom parameters constrained
Δρ_max_ = 0.38 e Å^−3^
Δρ_min_ = −0.40 e Å^−3^

### 

Data collection: *APEX2* (Bruker, 2009 ▶); cell refinement: *SAINT* (Bruker, 2009 ▶); data reduction: *SAINT*; program(s) used to solve structure: *SHELXS97* (Sheldrick, 2008 ▶); program(s) used to refine structure: *SHELXL97* (Sheldrick, 2008 ▶); molecular graphics: *ORTEP-3 for Windows* (Farrugia, 1997 ▶) and *PLATON* (Spek, 2009 ▶); software used to prepare material for publication: *WinGX* (Farrugia, 1999 ▶) and *PLATON*.

## Supplementary Material

Crystal structure: contains datablocks global, I. DOI: 10.1107/S1600536811011391/gk2358sup1.cif

Structure factors: contains datablocks I. DOI: 10.1107/S1600536811011391/gk2358Isup2.hkl

Additional supplementary materials:  crystallographic information; 3D view; checkCIF report

## Figures and Tables

**Fig. 1.**
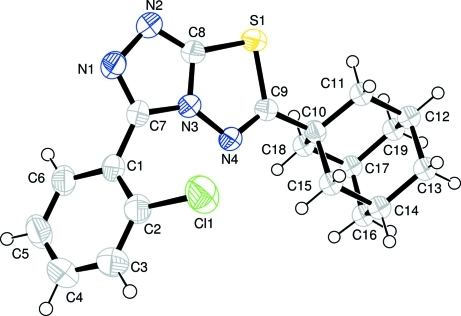
View of of the title compound with displacement ellipsoids drawn at the 50% probability level. H-atoms are shown by small spheres of arbitrary radius. Only one of the four disordered positions of the adamantyl group is shown.

## supplementary crystallographic information

### Comment

The crystal structure of the fluoro analogue of the title compound has been published (Khan *et al.*, 2009). The title molecule is shown in Fig. 1. The adamantyl group is disordered over four positions. The chlorobenzene moiety A (C1—C6/CL1) is planar with r. m. s. deviation of 0.0149 Å, whereas two fused heterocyclic rings B (C7/N1/N2/C8/S1/C9/N4/N3) are also planar with r. m. s. deviation of 0.0166 Å. The dihedral angle beteen A/B is 55.76 (6)°. The value of this dihedral angle in the fluoro isomer is 48.98 (6)°. There exist π—π interaction with the benzene ring centroids at a distance of 3.9526 (18) Å ( symmetry code: i = 1 - *x*, -*y*, 1 - *z)*. The crystal structure is mainly stabilized by van der Waals forces.

### Experimental

The title compound was prepared from a mixture of 4-amino-5-(2-chlorophenyl)-2*H*-1,2,4-triazole-3(4*H*)-thione and adamantane-1-carboxylic acid using the method described by Khan *et al.* (2009).

### Refinement

The adamantyl group is disordered. It has been modelled in four positions related by rotation around C-C bond to the thiadiazole ring. All atoms of the adamantyl group have been refined isotropically, with a common displacement parameter for the C atoms of each position. Restraints were imposed on 1-2 and 1-3 distances in the adamatyl ring [1.535 (5) and 2.50 (1) Å, respectively]. Equal occupancy factor of 0.25 was assumed for all  disordered positions.

The H-atoms were positioned geometrically (C–H = 0.93–0.98 Å) and treated as riding with *U*_iso_(H) = *xU*_eq_(C), where *x* = 1.2 for all H-atoms.

### Crystal data

**Table:**

C_19_H_19_ClN_4_S	*F*(000) = 1552
*M**_r_* = 370.89	*D*_x_ = 1.387 Mg m^−^^3^
Monoclinic, *C*2/*c*	Mo *K*α radiation, λ = 0.71073 Å
Hall symbol: -C 2yc	Cell parameters from 2676 reflections
*a* = 24.5210 (12) Å	θ = 2.5–25.3°
*b* = 9.1356 (5) Å	µ = 0.34 mm^−^^1^
*c* = 19.1943 (16) Å	*T* = 296 K
β = 124.282 (1)°	Prism, colorless
*V* = 3552.8 (4) Å^3^	0.28 × 0.24 × 0.20 mm
*Z* = 8	

### Data collection

**Table:**

Bruker Kappa APEXII CCD diffractometer	3202 independent reflections
Radiation source: fine-focus sealed tube	2676 reflections with *I* > 2σ(*I*)
graphite	*R*_int_ = 0.025
Detector resolution: 8.20 pixels mm^-1^	θ_max_ = 25.3°, θ_min_ = 2.5°
ω scans	*h* = −29→29
Absorption correction: multi-scan (*SADABS*; Bruker, 2005)	*k* = −10→10
*T*_min_ = 0.945, *T*_max_ = 0.956	*l* = −23→19
16675 measured reflections	

### Refinement

**Table:**

Refinement on *F*^2^	Primary atom site location: structure-invariant direct methods
Least-squares matrix: full	Secondary atom site location: difference Fourier map
*R*[*F*^2^ > 2σ(*F*^2^)] = 0.048	Hydrogen site location: inferred from neighbouring sites
*wR*(*F*^2^) = 0.125	H-atom parameters constrained
*S* = 1.02	*w* = 1/[σ^2^(*F*_o_^2^) + (0.0489*P*)^2^ + 7.1182*P*] where *P* = (*F*_o_^2^ + 2*F*_c_^2^)/3
3202 reflections	(Δ/σ)_max_ < 0.001
257 parameters	Δρ_max_ = 0.38 e Å^−^^3^
84 restraints	Δρ_min_ = −0.39 e Å^−^^3^

### Special details

**Table:**

Geometry. All esds (except the esd in the dihedral angle between two l.s. planes) are estimated using the full covariance matrix. The cell esds are taken into account individually in the estimation of esds in distances, angles and torsion angles; correlations between esds in cell parameters are only used when they are defined by crystal symmetry. An approximate (isotropic) treatment of cell esds is used for estimating esds involving l.s. planes.
Refinement. Refinement of *F*^2^ against ALL reflections. The weighted *R*-factor *wR* and goodness of fit *S* are based on *F*^2^, conventional *R*-factors *R* are based on *F*, with *F* set to zero for negative *F*^2^. The threshold expression of *F*^2^ > σ(*F*^2^) is used only for calculating *R*-factors(gt) *etc*. and is not relevant to the choice of reflections for refinement. *R*-factors based on *F*^2^ are statistically about twice as large as those based on *F*, and *R*- factors based on ALL data will be even larger.

### Fractional atomic coordinates and isotropic or equivalent isotropic displacement parameters (Å^2^)

**Table:**

	*x*	*y*	*z*	*U*_iso_*/*U*_eq_	Occ. (<1)
Cl1	0.42498 (4)	−0.09216 (11)	0.63099 (6)	0.0770 (3)	
S1	0.22506 (3)	−0.34790 (7)	0.36430 (5)	0.0495 (2)	
N1	0.40695 (10)	−0.4767 (2)	0.50704 (13)	0.0461 (5)	
N2	0.34112 (10)	−0.5233 (2)	0.45569 (14)	0.0489 (5)	
N3	0.34555 (9)	−0.2822 (2)	0.45879 (12)	0.0368 (4)	
N4	0.31664 (9)	−0.1464 (2)	0.43225 (12)	0.0396 (5)	
C1	0.46844 (11)	−0.2418 (3)	0.54892 (15)	0.0401 (5)	
C2	0.48003 (13)	−0.1275 (3)	0.60326 (17)	0.0491 (6)	
C3	0.53567 (14)	−0.0420 (3)	0.6378 (2)	0.0650 (8)	
H3	0.5428	0.0344	0.6741	0.078*	
C4	0.58072 (14)	−0.0703 (4)	0.6184 (2)	0.0696 (9)	
H4	0.6180	−0.0117	0.6408	0.083*	
C5	0.57103 (14)	−0.1844 (4)	0.5661 (2)	0.0637 (8)	
H5	0.6021	−0.2040	0.5540	0.076*	
C6	0.51534 (12)	−0.2698 (3)	0.53154 (17)	0.0500 (6)	
H6	0.5090	−0.3471	0.4962	0.060*	
C7	0.40903 (11)	−0.3331 (3)	0.50811 (14)	0.0382 (5)	
C8	0.30660 (12)	−0.4025 (3)	0.42871 (15)	0.0405 (5)	
C9	0.25384 (11)	−0.1642 (3)	0.38309 (15)	0.0386 (5)	
C10	0.20638 (10)	−0.0380 (2)	0.34271 (13)	0.0377 (5)	
C11	0.1416 (3)	−0.0728 (7)	0.3342 (6)	0.0373 (9)*	0.25
H11A	0.1213	−0.1588	0.2992	0.045*	0.25
H11B	0.1507	−0.0935	0.3894	0.045*	0.25
C12	0.0944 (3)	0.0576 (7)	0.2947 (6)	0.0373 (9)*	0.25
H12A	0.0523	0.0341	0.2868	0.045*	0.25
C13	0.1255 (4)	0.1924 (9)	0.3516 (6)	0.0373 (9)*	0.25
H13A	0.1319	0.1733	0.4055	0.045*	0.25
H13B	0.0958	0.2751	0.3258	0.045*	0.25
C14	0.1920 (3)	0.2299 (7)	0.3657 (4)	0.0373 (9)*	0.25
H14A	0.2120	0.3150	0.4029	0.045*	0.25
C15	0.2375 (3)	0.0958 (6)	0.4042 (4)	0.0373 (9)*	0.25
H15A	0.2442	0.0713	0.4576	0.045*	0.25
H15B	0.2802	0.1187	0.4147	0.045*	0.25
C16	0.1821 (6)	0.2592 (12)	0.2807 (6)	0.0373 (9)*	0.25
H16A	0.1554	0.3464	0.2554	0.045*	0.25
H16B	0.2246	0.2766	0.2895	0.045*	0.25
C17	0.1489 (4)	0.1321 (10)	0.2219 (5)	0.0373 (9)*	0.25
H17A	0.1409	0.1563	0.1671	0.045*	0.25
C18	0.1966 (5)	0.0022 (10)	0.2603 (4)	0.0373 (9)*	0.25
H18A	0.1787	−0.0805	0.2220	0.045*	0.25
H18B	0.2386	0.0284	0.2697	0.045*	0.25
C19	0.0834 (4)	0.0920 (13)	0.2096 (5)	0.0373 (9)*	0.25
H19A	0.0644	0.0075	0.1728	0.045*	0.25
H19B	0.0527	0.1729	0.1830	0.045*	0.25
C21	0.1331 (2)	−0.0855 (7)	0.2940 (6)	0.0375 (9)*	0.25
H21A	0.1236	−0.1540	0.2499	0.045*	0.25
H21B	0.1252	−0.1350	0.3323	0.045*	0.25
C22	0.0868 (3)	0.0464 (8)	0.2548 (6)	0.0375 (9)*	0.25
H22	0.0409	0.0136	0.2251	0.045*	0.25
C23	0.1012 (4)	0.1573 (10)	0.3227 (6)	0.0375 (9)*	0.25
H23A	0.0938	0.1127	0.3626	0.045*	0.25
H23B	0.0719	0.2406	0.2972	0.045*	0.25
C24	0.1732 (4)	0.2079 (7)	0.3686 (4)	0.0375 (9)*	0.25
H24A	0.1827	0.2795	0.4121	0.045*	0.25
C25	0.2187 (4)	0.0749 (8)	0.4100 (4)	0.0375 (9)*	0.25
H25A	0.2108	0.0297	0.4492	0.045*	0.25
H25B	0.2644	0.1067	0.4414	0.045*	0.25
C26	0.1824 (5)	0.2796 (11)	0.3037 (7)	0.0375 (9)*	0.25
H26A	0.1518	0.3607	0.2769	0.045*	0.25
H26B	0.2269	0.3179	0.3319	0.045*	0.25
C27	0.1704 (4)	0.1704 (10)	0.2380 (6)	0.0375 (9)*	0.25
H27	0.1776	0.2165	0.1977	0.045*	0.25
C28	0.2171 (5)	0.0392 (9)	0.2801 (5)	0.0375 (9)*	0.25
H28A	0.2625	0.0726	0.3097	0.045*	0.25
H28B	0.2096	−0.0299	0.2372	0.045*	0.25
C29	0.0985 (4)	0.1168 (12)	0.1920 (5)	0.0375 (9)*	0.25
H29A	0.0686	0.1987	0.1643	0.045*	0.25
H29B	0.0896	0.0460	0.1491	0.045*	0.25
C31	0.1576 (5)	−0.0417 (11)	0.3677 (8)	0.0785 (15)*	0.25
H31A	0.1812	−0.0366	0.4286	0.094*	0.25
H31B	0.1330	−0.1328	0.3490	0.094*	0.25
C32	0.1099 (4)	0.0882 (12)	0.3274 (7)	0.0785 (15)*	0.25
H32	0.0791	0.0893	0.3447	0.094*	0.25
C33	0.1494 (6)	0.2305 (11)	0.3531 (9)	0.0785 (15)*	0.25
H33A	0.1751	0.2404	0.4139	0.094*	0.25
H33B	0.1192	0.3128	0.3288	0.094*	0.25
C34	0.1956 (5)	0.2334 (8)	0.3235 (8)	0.0785 (15)*	0.25
H34	0.2187	0.3274	0.3367	0.094*	0.25
C35	0.2448 (4)	0.1059 (7)	0.3665 (8)	0.0785 (15)*	0.25
H35A	0.2745	0.1042	0.3483	0.094*	0.25
H35B	0.2708	0.1187	0.4272	0.094*	0.25
C36	0.1556 (9)	0.202 (2)	0.2287 (8)	0.0785 (15)*	0.25
H36A	0.1244	0.2813	0.1993	0.094*	0.25
H36B	0.1854	0.2018	0.2106	0.094*	0.25
C37	0.1198 (6)	0.0651 (14)	0.2040 (7)	0.0785 (15)*	0.25
H37	0.0947	0.0539	0.1426	0.094*	0.25
C38	0.1697 (6)	−0.0608 (14)	0.2470 (3)	0.0785 (15)*	0.25
H38A	0.1470	−0.1543	0.2308	0.094*	0.25
H38B	0.2006	−0.0595	0.2307	0.094*	0.25
C39	0.0727 (5)	0.064 (2)	0.2324 (7)	0.0785 (15)*	0.25
H39A	0.0401	0.1412	0.2031	0.094*	0.25
H39B	0.0497	−0.0285	0.2175	0.094*	0.25
C41	0.1975 (4)	0.0257 (9)	0.4083 (4)	0.0441 (9)*	0.25
H41A	0.2398	0.0576	0.4569	0.053*	0.25
H41B	0.1797	−0.0480	0.4266	0.053*	0.25
C42	0.1501 (4)	0.1560 (8)	0.3699 (4)	0.0441 (9)*	0.25
H42	0.1439	0.1989	0.4118	0.053*	0.25
C43	0.1781 (5)	0.2705 (7)	0.3403 (6)	0.0441 (9)*	0.25
H43A	0.2207	0.3036	0.3879	0.053*	0.25
H43B	0.1487	0.3543	0.3175	0.053*	0.25
C44	0.1860 (4)	0.2064 (8)	0.2727 (6)	0.0441 (9)*	0.25
H44	0.2041	0.2811	0.2545	0.053*	0.25
C45	0.2336 (4)	0.0758 (7)	0.3108 (6)	0.0441 (9)*	0.25
H45A	0.2768	0.1087	0.3572	0.053*	0.25
H45B	0.2384	0.0320	0.2685	0.053*	0.25
C46	0.1191 (5)	0.1544 (13)	0.1969 (6)	0.0441 (9)*	0.25
H46A	0.0890	0.2368	0.1720	0.053*	0.25
H46B	0.1245	0.1134	0.1545	0.053*	0.25
C47	0.0902 (4)	0.0383 (9)	0.2250 (5)	0.0441 (9)*	0.25
H47	0.0472	0.0048	0.1770	0.053*	0.25
C48	0.1387 (3)	−0.0905 (9)	0.2653 (5)	0.0441 (9)*	0.25
H48A	0.1449	−0.1340	0.2241	0.053*	0.25
H48B	0.1206	−0.1647	0.2830	0.053*	0.25
C49	0.0839 (4)	0.1042 (13)	0.2933 (6)	0.0441 (9)*	0.25
H49A	0.0536	0.1864	0.2698	0.053*	0.25
H49B	0.0653	0.0315	0.3111	0.053*	0.25

### Atomic displacement parameters (Å^2^)

**Table:**

	*U*^11^	*U*^22^	*U*^33^	*U*^12^	*U*^13^	*U*^23^
Cl1	0.0670 (5)	0.0945 (7)	0.0839 (6)	−0.0140 (4)	0.0512 (5)	−0.0385 (5)
S1	0.0379 (3)	0.0375 (4)	0.0665 (4)	−0.0041 (3)	0.0254 (3)	0.0007 (3)
N1	0.0481 (12)	0.0389 (12)	0.0539 (13)	0.0067 (9)	0.0302 (10)	0.0041 (10)
N2	0.0478 (12)	0.0370 (11)	0.0633 (14)	0.0022 (9)	0.0321 (11)	0.0025 (10)
N3	0.0373 (10)	0.0314 (10)	0.0416 (10)	0.0017 (8)	0.0221 (9)	0.0017 (8)
N4	0.0365 (10)	0.0327 (10)	0.0468 (11)	0.0028 (8)	0.0218 (9)	0.0036 (9)
C1	0.0353 (12)	0.0388 (13)	0.0413 (12)	0.0075 (10)	0.0186 (10)	0.0054 (10)
C2	0.0433 (13)	0.0497 (15)	0.0520 (15)	0.0018 (11)	0.0254 (12)	−0.0045 (12)
C3	0.0523 (16)	0.0581 (18)	0.075 (2)	−0.0104 (14)	0.0298 (15)	−0.0187 (15)
C4	0.0445 (16)	0.067 (2)	0.087 (2)	−0.0107 (14)	0.0306 (16)	−0.0064 (17)
C5	0.0416 (15)	0.076 (2)	0.077 (2)	0.0046 (14)	0.0353 (15)	0.0031 (17)
C6	0.0409 (13)	0.0523 (16)	0.0546 (15)	0.0084 (12)	0.0255 (12)	0.0012 (12)
C7	0.0384 (12)	0.0385 (13)	0.0392 (12)	0.0061 (10)	0.0227 (10)	0.0020 (10)
C8	0.0414 (13)	0.0362 (12)	0.0482 (14)	−0.0020 (10)	0.0278 (11)	−0.0002 (11)
C9	0.0377 (12)	0.0350 (12)	0.0449 (13)	−0.0011 (10)	0.0244 (11)	0.0014 (10)
C10	0.0327 (11)	0.0374 (12)	0.0427 (12)	0.0016 (9)	0.0210 (10)	0.0038 (10)

### Geometric parameters (Å, °)

**Table:**

Cl1—C2	1.737 (3)	C23—H23B	0.9700
S1—C8	1.729 (2)	C24—C26	1.533 (5)
S1—C9	1.777 (2)	C24—C25	1.534 (5)
N1—C7	1.313 (3)	C24—H24A	0.9800
N1—N2	1.402 (3)	C25—H25A	0.9700
N2—C8	1.307 (3)	C25—H25B	0.9700
N3—C8	1.354 (3)	C26—C27	1.502 (12)
N3—C7	1.369 (3)	C26—H26A	0.9700
N3—N4	1.376 (3)	C26—H26B	0.9700
N4—C9	1.285 (3)	C27—C28	1.536 (5)
C1—C2	1.388 (4)	C27—C29	1.541 (5)
C1—C6	1.390 (3)	C27—H27	0.9800
C1—C7	1.465 (3)	C28—H28A	0.9700
C2—C3	1.376 (4)	C28—H28B	0.9700
C3—C4	1.375 (4)	C29—H29A	0.9700
C3—H3	0.9300	C29—H29B	0.9700
C4—C5	1.372 (5)	C31—C32	1.535 (5)
C4—H4	0.9300	C31—H31A	0.9700
C5—C6	1.376 (4)	C31—H31B	0.9700
C5—H5	0.9300	C32—C39	1.527 (5)
C6—H6	0.9300	C32—C33	1.528 (5)
C9—C10	1.506 (3)	C32—H32	0.9800
C10—C18	1.506 (4)	C33—C34	1.528 (5)
C10—C41	1.510 (4)	C33—H33A	0.9700
C10—C31	1.520 (5)	C33—H33B	0.9700
C10—C35	1.529 (5)	C34—C36	1.530 (5)
C10—C11	1.536 (4)	C34—C35	1.540 (5)
C10—C45	1.537 (4)	C34—H34	0.9800
C10—C38	1.537 (5)	C35—H35A	0.9700
C10—C28	1.538 (4)	C35—H35B	0.9700
C10—C25	1.543 (4)	C36—C37	1.447 (19)
C10—C21	1.549 (4)	C36—H36A	0.9700
C10—C48	1.553 (4)	C36—H36B	0.9700
C11—C12	1.531 (5)	C37—C39	1.529 (5)
C11—H11A	0.9700	C37—C38	1.537 (5)
C11—H11B	0.9700	C37—H37	0.9800
C12—C19	1.532 (5)	C38—H38A	0.9700
C12—C13	1.533 (5)	C38—H38B	0.9700
C12—H12A	0.9800	C39—H39A	0.9700
C13—C14	1.534 (5)	C39—H39B	0.9700
C13—H13A	0.9700	C41—C42	1.532 (5)
C13—H13B	0.9700	C41—H41A	0.9700
C14—C16	1.533 (5)	C41—H41B	0.9700
C14—C15	1.538 (5)	C42—C43	1.525 (5)
C14—H14A	0.9800	C42—C49	1.527 (5)
C15—H15A	0.9700	C42—H42	0.9800
C15—H15B	0.9700	C43—C44	1.533 (5)
C16—C17	1.500 (13)	C43—H43A	0.9700
C16—H16A	0.9700	C43—H43B	0.9700
C16—H16B	0.9700	C44—C46	1.532 (5)
C17—C19	1.531 (5)	C44—C45	1.537 (5)
C17—C18	1.532 (5)	C44—H44	0.9800
C17—H17A	0.9800	C45—H45A	0.9700
C18—H18A	0.9700	C45—H45B	0.9700
C18—H18B	0.9700	C46—C47	1.531 (14)
C19—H19A	0.9700	C46—H46A	0.9700
C19—H19B	0.9700	C46—H46B	0.9700
C21—C22	1.531 (5)	C47—C49	1.528 (5)
C21—H21A	0.9700	C47—C48	1.537 (5)
C21—H21B	0.9700	C47—H47	0.9800
C22—C23	1.527 (5)	C48—H48A	0.9700
C22—C29	1.529 (5)	C48—H48B	0.9700
C22—H22	0.9800	C49—H49A	0.9700
C23—C24	1.534 (5)	C49—H49B	0.9700
C23—H23A	0.9700		
			
C8—S1—C9	87.73 (11)	C24—C25—H25B	109.5
C7—N1—N2	109.47 (19)	C10—C25—H25B	109.5
C8—N2—N1	104.77 (19)	H25A—C25—H25B	108.1
C8—N3—C7	105.86 (19)	C27—C26—C24	110.5 (7)
C8—N3—N4	118.77 (18)	C27—C26—H26A	109.6
C7—N3—N4	135.24 (19)	C24—C26—H26A	109.6
C9—N4—N3	108.23 (19)	C27—C26—H26B	109.6
C2—C1—C6	118.2 (2)	C24—C26—H26B	109.6
C2—C1—C7	123.4 (2)	H26A—C26—H26B	108.1
C6—C1—C7	118.4 (2)	C26—C27—C28	109.9 (7)
C3—C2—C1	121.2 (3)	C26—C27—C29	108.2 (7)
C3—C2—Cl1	118.5 (2)	C28—C27—C29	109.1 (7)
C1—C2—Cl1	120.3 (2)	C26—C27—H27	109.9
C4—C3—C2	119.5 (3)	C28—C27—H27	109.9
C4—C3—H3	120.3	C29—C27—H27	109.9
C2—C3—H3	120.3	C27—C28—C10	111.4 (5)
C5—C4—C3	120.5 (3)	C27—C28—H28A	109.3
C5—C4—H4	119.8	C10—C28—H28A	109.3
C3—C4—H4	119.8	C27—C28—H28B	109.3
C4—C5—C6	119.9 (3)	C10—C28—H28B	109.3
C4—C5—H5	120.0	H28A—C28—H28B	108.0
C6—C5—H5	120.0	C22—C29—C27	110.1 (5)
C5—C6—C1	120.7 (3)	C22—C29—H29A	109.6
C5—C6—H6	119.6	C27—C29—H29A	109.6
C1—C6—H6	119.6	C22—C29—H29B	109.6
N1—C7—N3	108.0 (2)	C27—C29—H29B	109.6
N1—C7—C1	126.5 (2)	H29A—C29—H29B	108.2
N3—C7—C1	125.3 (2)	C10—C31—C32	109.6 (5)
N2—C8—N3	111.9 (2)	C10—C31—H31A	109.7
N2—C8—S1	139.2 (2)	C32—C31—H31A	109.7
N3—C8—S1	108.93 (17)	C10—C31—H31B	109.7
N4—C9—C10	122.7 (2)	C32—C31—H31B	109.7
N4—C9—S1	116.27 (17)	H31A—C31—H31B	108.2
C10—C9—S1	121.00 (16)	C39—C32—C33	110.4 (10)
C9—C10—C18	110.3 (3)	C39—C32—C31	105.3 (10)
C9—C10—C41	107.4 (3)	C33—C32—C31	109.3 (6)
C9—C10—C31	109.8 (4)	C39—C32—H32	110.5
C9—C10—C35	109.7 (4)	C33—C32—H32	110.5
C31—C10—C35	112.8 (5)	C31—C32—H32	110.5
C9—C10—C11	110.5 (3)	C32—C33—C34	111.3 (6)
C18—C10—C11	113.4 (4)	C32—C33—H33A	109.4
C9—C10—C45	109.8 (3)	C34—C33—H33A	109.4
C41—C10—C45	111.7 (4)	C32—C33—H33B	109.4
C9—C10—C38	105.9 (5)	C34—C33—H33B	109.4
C31—C10—C38	109.8 (5)	H33A—C33—H33B	108.0
C35—C10—C38	108.7 (5)	C33—C34—C36	109.0 (10)
C9—C10—C28	109.3 (3)	C33—C34—C35	108.0 (6)
C9—C10—C25	110.5 (3)	C36—C34—C35	106.3 (10)
C28—C10—C25	107.9 (4)	C33—C34—H34	111.1
C9—C10—C21	113.2 (3)	C36—C34—H34	111.1
C28—C10—C21	108.0 (4)	C35—C34—H34	111.1
C25—C10—C21	107.8 (4)	C10—C35—C34	109.0 (5)
C9—C10—C48	110.8 (3)	C10—C35—H35A	109.9
C41—C10—C48	109.8 (4)	C34—C35—H35A	109.9
C45—C10—C48	107.3 (4)	C10—C35—H35B	109.9
C12—C11—C10	109.9 (4)	C34—C35—H35B	109.9
C12—C11—H11A	109.7	H35A—C35—H35B	108.3
C10—C11—H11A	109.7	C37—C36—C34	114.5 (11)
C12—C11—H11B	109.7	C37—C36—H36A	108.6
C10—C11—H11B	109.7	C34—C36—H36A	108.6
H11A—C11—H11B	108.2	C37—C36—H36B	108.6
C11—C12—C19	108.5 (7)	C34—C36—H36B	108.6
C11—C12—C13	109.8 (5)	H36A—C36—H36B	107.6
C19—C12—C13	108.3 (7)	C36—C37—C39	109.1 (11)
C11—C12—H12A	110.1	C36—C37—C38	108.6 (11)
C19—C12—H12A	110.1	C39—C37—C38	109.7 (11)
C13—C12—H12A	110.1	C36—C37—H37	109.8
C12—C13—C14	110.9 (5)	C39—C37—H37	109.8
C12—C13—H13A	109.5	C38—C37—H37	109.8
C14—C13—H13A	109.5	C10—C38—C37	107.1 (7)
C12—C13—H13B	109.5	C10—C38—H38A	110.3
C14—C13—H13B	109.5	C37—C38—H38A	110.3
H13A—C13—H13B	108.0	C10—C38—H38B	110.3
C16—C14—C13	109.7 (7)	C37—C38—H38B	110.3
C16—C14—C15	106.7 (7)	H38A—C38—H38B	108.6
C13—C14—C15	108.6 (5)	C32—C39—C37	111.3 (7)
C16—C14—H14A	110.6	C32—C39—H39A	109.4
C13—C14—H14A	110.6	C37—C39—H39A	109.4
C15—C14—H14A	110.6	C32—C39—H39B	109.4
C14—C15—C10	110.2 (4)	C37—C39—H39B	109.4
C14—C15—H15A	109.6	H39A—C39—H39B	108.0
C10—C15—H15A	109.6	C10—C41—C42	109.0 (4)
C14—C15—H15B	109.6	C10—C41—H41A	109.9
C10—C15—H15B	109.6	C42—C41—H41A	109.9
H15A—C15—H15B	108.1	C10—C41—H41B	109.9
C17—C16—C14	111.0 (7)	C42—C41—H41B	109.9
C17—C16—H16A	109.4	H41A—C41—H41B	108.3
C14—C16—H16A	109.4	C43—C42—C49	108.2 (7)
C17—C16—H16B	109.4	C43—C42—C41	109.5 (5)
C14—C16—H16B	109.4	C49—C42—C41	109.2 (7)
H16A—C16—H16B	108.0	C43—C42—H42	110.0
C16—C17—C19	112.3 (7)	C49—C42—H42	110.0
C16—C17—C18	107.1 (8)	C41—C42—H42	110.0
C19—C17—C18	109.9 (7)	C42—C43—C44	110.6 (5)
C16—C17—H17A	109.2	C42—C43—H43A	109.5
C19—C17—H17A	109.2	C44—C43—H43A	109.5
C18—C17—H17A	109.2	C42—C43—H43B	109.5
C10—C18—C17	108.7 (5)	C44—C43—H43B	109.5
C10—C18—H18A	109.9	H43A—C43—H43B	108.1
C17—C18—H18A	109.9	C46—C44—C43	110.1 (7)
C10—C18—H18B	109.9	C46—C44—C45	109.4 (7)
C17—C18—H18B	109.9	C43—C44—C45	108.6 (5)
H18A—C18—H18B	108.3	C46—C44—H44	109.6
C17—C19—C12	110.2 (5)	C43—C44—H44	109.6
C17—C19—H19A	109.6	C45—C44—H44	109.6
C12—C19—H19A	109.6	C44—C45—C10	109.3 (4)
C17—C19—H19B	109.6	C44—C45—H45A	109.8
C12—C19—H19B	109.6	C10—C45—H45A	109.8
H19A—C19—H19B	108.1	C44—C45—H45B	109.8
C22—C21—C10	111.3 (4)	C10—C45—H45B	109.8
C22—C21—H21A	109.4	H45A—C45—H45B	108.3
C10—C21—H21A	109.4	C47—C46—C44	109.9 (7)
C22—C21—H21B	109.4	C47—C46—H46A	109.7
C10—C21—H21B	109.4	C44—C46—H46A	109.7
H21A—C21—H21B	108.0	C47—C46—H46B	109.7
C23—C22—C29	109.8 (7)	C44—C46—H46B	109.7
C23—C22—C21	110.5 (5)	H46A—C46—H46B	108.2
C29—C22—C21	107.7 (7)	C49—C47—C46	108.7 (7)
C23—C22—H22	109.6	C49—C47—C48	107.1 (8)
C29—C22—H22	109.6	C46—C47—C48	109.1 (8)
C21—C22—H22	109.6	C49—C47—H47	110.6
C22—C23—C24	109.1 (5)	C46—C47—H47	110.6
C22—C23—H23A	109.9	C48—C47—H47	110.6
C24—C23—H23A	109.9	C47—C48—C10	110.6 (5)
C22—C23—H23B	109.9	C47—C48—H48A	109.5
C24—C23—H23B	109.9	C10—C48—H48A	109.5
H23A—C23—H23B	108.3	C47—C48—H48B	109.5
C26—C24—C25	111.2 (7)	C10—C48—H48B	109.5
C26—C24—C23	108.1 (7)	H48A—C48—H48B	108.1
C25—C24—C23	109.0 (5)	C42—C49—C47	112.4 (6)
C26—C24—H24A	109.5	C42—C49—H49A	109.1
C25—C24—H24A	109.5	C47—C49—H49A	109.1
C23—C24—H24A	109.5	C42—C49—H49B	109.1
C24—C25—C10	110.7 (4)	C47—C49—H49B	109.1
C24—C25—H25A	109.5	H49A—C49—H49B	107.9
C10—C25—H25A	109.5		
			
C7—N1—N2—C8	−0.4 (3)	C31—C10—C25—C24	−80.5 (6)
C8—N3—N4—C9	2.4 (3)	C35—C10—C25—C24	77.4 (7)
C7—N3—N4—C9	177.5 (2)	C11—C10—C25—C24	−72.9 (6)
C6—C1—C2—C3	1.3 (4)	C45—C10—C25—C24	66.5 (6)
C7—C1—C2—C3	−177.9 (3)	C38—C10—C25—C24	9.1 (12)
C6—C1—C2—Cl1	−177.2 (2)	C28—C10—C25—C24	57.3 (6)
C7—C1—C2—Cl1	3.6 (4)	C21—C10—C25—C24	−59.1 (6)
C1—C2—C3—C4	−0.1 (5)	C48—C10—C25—C24	−43.5 (8)
Cl1—C2—C3—C4	178.4 (3)	C25—C24—C26—C27	56.9 (9)
C2—C3—C4—C5	−1.1 (5)	C23—C24—C26—C27	−62.7 (9)
C3—C4—C5—C6	1.1 (5)	C24—C26—C27—C28	−57.4 (9)
C4—C5—C6—C1	0.1 (5)	C24—C26—C27—C29	61.6 (9)
C2—C1—C6—C5	−1.3 (4)	C26—C27—C28—C10	60.1 (9)
C7—C1—C6—C5	177.9 (2)	C29—C27—C28—C10	−58.4 (9)
N2—N1—C7—N3	0.5 (3)	C9—C10—C28—C27	−179.2 (6)
N2—N1—C7—C1	−175.0 (2)	C18—C10—C28—C27	84.3 (15)
C8—N3—C7—N1	−0.3 (3)	C41—C10—C28—C27	−44.3 (9)
N4—N3—C7—N1	−175.9 (2)	C31—C10—C28—C27	12.3 (12)
C8—N3—C7—C1	175.2 (2)	C35—C10—C28—C27	−77.2 (7)
N4—N3—C7—C1	−0.3 (4)	C11—C10—C28—C27	40.8 (9)
C2—C1—C7—N1	−129.4 (3)	C45—C10—C28—C27	−83.9 (12)
C6—C1—C7—N1	51.5 (4)	C38—C10—C28—C27	85.6 (9)
C2—C1—C7—N3	55.9 (4)	C25—C10—C28—C27	−59.0 (7)
C6—C1—C7—N3	−123.3 (3)	C21—C10—C28—C27	57.3 (7)
N1—N2—C8—N3	0.2 (3)	C48—C10—C28—C27	69.6 (7)
N1—N2—C8—S1	179.3 (2)	C23—C22—C29—C27	58.9 (9)
C7—N3—C8—N2	0.1 (3)	C21—C22—C29—C27	−61.5 (9)
N4—N3—C8—N2	176.5 (2)	C26—C27—C29—C22	−59.3 (9)
C7—N3—C8—S1	−179.28 (15)	C28—C27—C29—C22	60.2 (9)
N4—N3—C8—S1	−2.9 (3)	C9—C10—C31—C32	179.2 (6)
C9—S1—C8—N2	−177.2 (3)	C18—C10—C31—C32	−38.4 (10)
C9—S1—C8—N3	1.85 (18)	C41—C10—C31—C32	85.4 (8)
N3—N4—C9—C10	179.52 (19)	C35—C10—C31—C32	56.6 (9)
N3—N4—C9—S1	−0.7 (3)	C11—C10—C31—C32	−84.8 (14)
C8—S1—C9—N4	−0.7 (2)	C45—C10—C31—C32	21.6 (12)
C8—S1—C9—C10	179.10 (19)	C38—C10—C31—C32	−64.7 (9)
N4—C9—C10—C18	87.7 (5)	C28—C10—C31—C32	−12.4 (13)
S1—C9—C10—C18	−92.1 (5)	C25—C10—C31—C32	75.3 (7)
N4—C9—C10—C41	−79.5 (5)	C21—C10—C31—C32	−76.9 (8)
S1—C9—C10—C41	100.7 (4)	C48—C10—C31—C32	−74.5 (8)
N4—C9—C10—C31	−122.0 (6)	C10—C31—C32—C39	62.7 (10)
S1—C9—C10—C31	58.3 (6)	C10—C31—C32—C33	−56.0 (10)
N4—C9—C10—C35	2.5 (6)	C39—C32—C33—C34	−55.0 (11)
S1—C9—C10—C35	−177.3 (6)	C31—C32—C33—C34	60.4 (11)
N4—C9—C10—C11	−146.2 (5)	C32—C33—C34—C36	53.4 (12)
S1—C9—C10—C11	34.0 (5)	C32—C33—C34—C35	−61.7 (11)
N4—C9—C10—C45	42.2 (5)	C9—C10—C35—C34	179.0 (6)
S1—C9—C10—C45	−137.6 (4)	C18—C10—C35—C34	71.2 (8)
N4—C9—C10—C38	119.5 (6)	C41—C10—C35—C34	−76.9 (7)
S1—C9—C10—C38	−60.2 (6)	C31—C10—C35—C34	−58.3 (9)
N4—C9—C10—C28	65.9 (5)	C11—C10—C35—C34	−40.1 (11)
S1—C9—C10—C28	−113.9 (5)	C45—C10—C35—C34	81.9 (10)
N4—C9—C10—C25	−52.7 (5)	C38—C10—C35—C34	63.7 (9)
S1—C9—C10—C25	127.5 (4)	C28—C10—C35—C34	77.7 (8)
N4—C9—C10—C21	−173.8 (5)	C25—C10—C35—C34	−80.0 (7)
S1—C9—C10—C21	6.5 (5)	C21—C10—C35—C34	−6.1 (12)
N4—C9—C10—C48	160.6 (5)	C48—C10—C35—C34	28.1 (11)
S1—C9—C10—C48	−19.2 (5)	C33—C34—C35—C10	58.8 (10)
C9—C10—C11—C12	178.9 (5)	C36—C34—C35—C10	−58.1 (11)
C18—C10—C11—C12	−56.8 (7)	C33—C34—C36—C37	−57.0 (15)
C41—C10—C11—C12	79.3 (6)	C35—C34—C36—C37	59.2 (15)
C31—C10—C11—C12	86.4 (12)	C34—C36—C37—C39	58.3 (16)
C35—C10—C11—C12	38.2 (9)	C34—C36—C37—C38	−61.3 (15)
C45—C10—C11—C12	−13.2 (10)	C9—C10—C38—C37	178.9 (8)
C38—C10—C11—C12	−74.8 (7)	C18—C10—C38—C37	−77.9 (12)
C28—C10—C11—C12	−41.5 (8)	C41—C10—C38—C37	29.2 (14)
C25—C10—C11—C12	68.4 (6)	C31—C10—C38—C37	60.5 (10)
C21—C10—C11—C12	−79.9 (9)	C35—C10—C38—C37	−63.3 (10)
C48—C10—C11—C12	−80.7 (7)	C11—C10—C38—C37	68.1 (9)
C10—C11—C12—C19	56.9 (7)	C45—C10—C38—C37	−74.7 (9)
C10—C11—C12—C13	−61.3 (8)	C28—C10—C38—C37	−78.8 (9)
C11—C12—C13—C14	58.0 (8)	C25—C10—C38—C37	−13.2 (15)
C19—C12—C13—C14	−60.4 (8)	C21—C10—C38—C37	70.6 (9)
C12—C13—C14—C16	58.8 (8)	C48—C10—C38—C37	75.3 (10)
C12—C13—C14—C15	−57.4 (8)	C36—C37—C38—C10	60.9 (11)
C16—C14—C15—C10	−57.6 (8)	C39—C37—C38—C10	−58.3 (11)
C13—C14—C15—C10	60.6 (7)	C33—C32—C39—C37	56.3 (15)
C9—C10—C15—C14	178.8 (5)	C31—C32—C39—C37	−61.6 (15)
C18—C10—C15—C14	59.4 (6)	C36—C37—C39—C32	−57.2 (17)
C41—C10—C15—C14	−83.6 (6)	C38—C37—C39—C32	61.6 (15)
C31—C10—C15—C14	−71.9 (6)	C9—C10—C41—C42	179.3 (5)
C35—C10—C15—C14	79.6 (9)	C18—C10—C41—C42	18.2 (10)
C11—C10—C15—C14	−62.9 (6)	C31—C10—C41—C42	−80.4 (8)
C45—C10—C15—C14	73.9 (6)	C35—C10—C41—C42	72.4 (6)
C38—C10—C15—C14	41.7 (9)	C11—C10—C41—C42	−76.2 (6)
C28—C10—C15—C14	68.5 (6)	C45—C10—C41—C42	58.8 (7)
C25—C10—C15—C14	−79.6 (11)	C38—C10—C41—C42	−31.2 (11)
C21—C10—C15—C14	−44.0 (8)	C28—C10—C41—C42	43.7 (8)
C48—C10—C15—C14	−17.8 (10)	C25—C10—C41—C42	77.9 (9)
C13—C14—C16—C17	−55.4 (9)	C21—C10—C41—C42	−67.3 (6)
C15—C14—C16—C17	62.1 (9)	C48—C10—C41—C42	−60.2 (7)
C14—C16—C17—C19	55.4 (10)	C10—C41—C42—C43	−58.9 (8)
C14—C16—C17—C18	−65.3 (9)	C10—C41—C42—C49	59.4 (7)
C9—C10—C18—C17	−179.2 (5)	C49—C42—C43—C44	−58.1 (8)
C41—C10—C18—C17	−18.5 (11)	C41—C42—C43—C44	60.9 (8)
C31—C10—C18—C17	38.6 (10)	C42—C43—C44—C46	59.5 (9)
C35—C10—C18—C17	−72.1 (7)	C42—C43—C44—C45	−60.2 (8)
C11—C10—C18—C17	56.4 (7)	C46—C44—C45—C10	−62.2 (8)
C45—C10—C18—C17	−81.6 (7)	C43—C44—C45—C10	57.9 (8)
C38—C10—C18—C17	93.9 (12)	C9—C10—C45—C44	−177.9 (5)
C28—C10—C18—C17	−88.2 (14)	C18—C10—C45—C44	83.3 (8)
C25—C10—C18—C17	−45.1 (8)	C41—C10—C45—C44	−58.9 (7)
C21—C10—C18—C17	66.2 (7)	C31—C10—C45—C44	−20.3 (11)
C48—C10—C18—C17	75.9 (7)	C35—C10—C45—C44	−81.2 (8)
C16—C17—C18—C10	64.3 (8)	C11—C10—C45—C44	14.1 (10)
C19—C17—C18—C10	−57.9 (9)	C38—C10—C45—C44	80.9 (7)
C16—C17—C19—C12	−57.3 (10)	C28—C10—C45—C44	89.4 (13)
C18—C17—C19—C12	61.8 (9)	C25—C10—C45—C44	−67.0 (6)
C11—C12—C19—C17	−60.7 (9)	C21—C10—C45—C44	43.0 (8)
C13—C12—C19—C17	58.4 (9)	C48—C10—C45—C44	61.5 (7)
C9—C10—C21—C22	179.6 (5)	C43—C44—C46—C47	−59.0 (10)
C18—C10—C21—C22	−68.5 (6)	C45—C44—C46—C47	60.2 (10)
C41—C10—C21—C22	71.9 (6)	C44—C46—C47—C49	57.9 (10)
C31—C10—C21—C22	83.3 (7)	C44—C46—C47—C48	−58.5 (10)
C35—C10—C21—C22	4.8 (11)	C49—C47—C48—C10	−57.9 (9)
C11—C10—C21—C22	90.3 (9)	C46—C47—C48—C10	59.5 (9)
C45—C10—C21—C22	−42.5 (8)	C9—C10—C48—C47	179.3 (6)
C38—C10—C21—C22	−83.9 (8)	C18—C10—C48—C47	−76.5 (7)
C28—C10—C21—C22	−59.3 (7)	C41—C10—C48—C47	60.9 (8)
C25—C10—C21—C22	57.0 (7)	C31—C10—C48—C47	74.5 (7)
C48—C10—C21—C22	−91.2 (12)	C35—C10—C48—C47	−29.9 (11)
C10—C21—C22—C23	−58.2 (8)	C11—C10—C48—C47	79.7 (8)
C10—C21—C22—C29	61.7 (8)	C45—C10—C48—C47	−60.8 (8)
C29—C22—C23—C24	−59.5 (7)	C38—C10—C48—C47	−90.9 (10)
C21—C22—C23—C24	59.1 (8)	C28—C10—C48—C47	−71.0 (7)
C22—C23—C24—C26	60.4 (8)	C25—C10—C48—C47	39.6 (9)
C22—C23—C24—C25	−60.6 (8)	C21—C10—C48—C47	78.9 (12)
C26—C24—C25—C10	−57.2 (8)	C43—C42—C49—C47	59.0 (10)
C23—C24—C25—C10	61.9 (8)	C41—C42—C49—C47	−60.1 (10)
C9—C10—C25—C24	176.7 (5)	C46—C47—C49—C42	−59.2 (11)
C18—C10—C25—C24	42.7 (8)	C48—C47—C49—C42	58.6 (10)
C41—C10—C25—C24	−95.8 (9)		
